# An adaptively multi-correlations aggregation network for skeleton-based motion recognition

**DOI:** 10.1038/s41598-023-46155-3

**Published:** 2023-11-06

**Authors:** Xinpeng Yin, Jianqi Zhong, Deliang Lian, Wenming Cao

**Affiliations:** 1 Guangdong Multimedia Information Service Engineering Technology Research Center, Shenzhen University, Yuehai Street, Shenzhen, 518060 China; 2 State Key Laboratory of Radio Frequency Heterogeneous Integration, Shenzhen University, Yuehai Street, Shenzhen, 51886 China

**Keywords:** Computational science, Computer science

## Abstract

Previous work based on Graph Convolutional Networks (GCNs) has shown promising performance in 3D skeleton-based motion recognition. We believe that the 3D skeleton-based motion recognition problem can be explained as a modeling task of dynamic skeleton-based graph construction. However, existing methods fail to model human poses with dynamic correlations between human joints, ignoring the information contained in the skeleton structure of the non-connected relationship during human motion modeling. In this paper, we propose an Adaptively Multi-correlations Aggregation Network(AMANet) to capture dynamic joint dependencies embedded in skeleton graphs, which includes three key modules: the Spatial Feature Extraction Module (SFEM), Temporal Feature Extraction Module (TFEM), and Spatio-Temporal Feature Extraction Module (STFEM). In addition, we deploy the relative coordinates of the joints of various parts of the human body via moving frames of Differential Geometry. On this basis, we design a Data Preprocessing Module (DP), enriching the characteristics of the original skeleton data. Extensive experiments are conducted on three public datasets(NTU-RGB+D 60, NTU-RGB+D 120, and Kinetics-Skeleton 400), demonstrating our proposed method’s effectiveness.

## Introduction

Skeleton-based action recognition aims to recognize human actions category given the complete action execution, which has become a research hotspot due to its comprehensive application scenarios in human-computer interaction^1, 2^, autonomous driving^3, 4^, video surveillance^5, 6^, and so on.

In the early stage, motion data based on RGB video, depth maps, and optical flow information are used as primary representations of human motion. Compared with these representations, human 3D skeleton data has the advantages of being free from the interference of video background information, eliminating the influence of external factors, and having a high degree of aggregation of sufficient data. These make it become the mainstream data format of human action recognition. With the maturity of pose estimation algorithms and the optimization of the depth camera technology^7, 8^, the calibration of 3D skeleton data has become more and more accurate, and the accuracy of researchers using skeleton data for action recognition analysis has also improved a lot.

The human skeleton is a natural graph structure, with joints as the points of the graph and bones as the edges. Generally, most GCNs-based models try to make contributions on the graph’s adjacency matrix for higher prediction performance. Where Yan et al.^9^ applied Graph Convolutional Networks (GCNs) for human skeleton-based action recognition. They used the graph’s adjacency matrix to aggregate the features of the human body’s connected joints and extend GCNs to ST-GCN to capture the flexible spatio-temporal relationship of motion dependencies. Li et al.^10^ proposed AS-GCN based on ST-GCN, compared with the limitation of ST-GCN that only refers to adjacent joints, the reference joint is extended to several adjacent joints to aggregate more information on the larger receptive field. Shi et al.^11^ presented 2S-AGCN to transform GCNs multiplications into additions, establishing latent relationships of non-adjacent joints. The MS-GCN module designed by Liu et al.^12^ solves the problem of biased weights in multi-scale adjacency matrices. The above improvements are limited to the inherent connection structure between joints, and an adjacency matrix $A\in \{0, 1\}^{N\times {N}}$ represents the structural relationship^13, 14^. A(i,j) = 1 indicates that the $i\textrm{th}$ joint and the $j\textrm{th}$ joint are adjacent, otherwise not. It is unreasonable that this alternative setting method makes the joints show strong correlation characteristics and irrelevant characteristics during the training process of the whole model. This is also why the GCN-based SOTA model suffers from over-smoothing issues when increasing depth^15^. As Chen et al.^16^ put forward, during the movement of the human body, different activities will change the correlation between joints, as in the action of clapping, the correlation between joints of the left and right hand is high. In the act of waving, the correlation between the two will weaken. Hence, expressing the correlation between the joints with the same adjacency matrix is inappropriate. We need a new method to represent the correlation between joints.

Recently, the success of Self-attention in natural language processing has shown that modeling with attention can help improve its recognition ability^17, 18^ effectively. This motivates us to develop an attention network based on skeleton data for human action recognition.

For human skeleton data, the correlation between joints can be obtained by the inner product of the eigenvectors of each joint. Obviously, the correlation is represented by an obliquely symmetric matrix, it is unified with the adjacency matrix in GCNs in terms of physical meaning and mathematical expression. The elements in the adjacency matrix are only 0 and 1, while in the correlation matrix are arbitrary values, which can more abundantly characterize the correlation between joints.

Based on the above observations, we integrate the GCNs method and Self-attention to design AMANet, which includes a Spatial Feature Extraction Module (SFEM), a Temporal Feature Extraction Module (TFEM), and a Spatio-Temporal Fusion Feature Extraction Module (STFEM). SFEM extracts the spatial features of the sequence according to the correlation between the joints in each frame, TFEM extracts the temporal features of the series according to the correlation between frames, and the STFEM aims to extract the spatio-temporal characteristics of adjacent multi-frames simultaneously. Furthermore, to extract richer features from limited data, we design a Data Preprocessing (DP) module^19, 20^. In this module, we obtain three independent spatio-temporal feature sequences from the original skeleton data, including the skeleton velocity sequence, the skeleton acceleration sequence, and the relative position sequence. As for the entire system, we adopt a Multi-Scale Dual-stream (MS-DS) architecture inspired by dual-stream network^21, 22^ and multi-scale^12^. The final softmax scores obtained by training joint and bone data at different scales are weighted and added to obtain the final recognition score.

The main contributions of this paper are summarized as follows:

1. To extract richer features from limited data, Data Pre-processing (DP) module is designed to include four independent spatio-temporal feature sequences, namely the original skeleton sequence, the skeleton velocity sequence, the skeleton acceleration sequence, and the relative position sequence.
2. Through unifying GCNs and Self-attention, we propose Adaptively Multi-correlations Aggregation Network (AMANet) to capture dynamic correlations between human joints when considering multi-scale feature aggregation.
3. In AMANet, we propose three key modules to learn rich motion representation: the Spatial Feature Extraction Module (SFEM) to capture motion dependencies in the spatial-wise channel, Temporal Feature Extraction Module (TFEM) to exploit temporal information for all motion joints, and Spatio-Temporal Feature Extraction module (STFEM) to comprehensively model motion dynamics.
4. We conduct extensive experiments on AMANet on three public datasets, including NTU-RGB+D 60, NTU-RGB+D 120, and Kinetics-Skeleton, which verify that our method is highly competitive compared to existing the SOTA models.

The rest of this paper is organized as follows: section “Related work” shows recent works related to our work, section “Method” describes explicitly several vital modules of our proposed architecture, section “Experiments” illustrates the details of our experiments, and section “Conclusion” concludes this work.

## Related work

### Early human action recognition

A key issue in human action recognition research is extracting distinguishable and rich features from limited data^23, 24^. Before the deep learning method was applied in this field, the dense trajectory algorithm (DT) and its improved version (IDT) algorithm^25, 26^ were excellent among the traditional human action recognition algorithms. The method collects feature points for each video frame and extracts the feature points’ trajectories in the time dimension. At the same time, it combines the motion descriptor of the feature points to supplement the description of the optical flow features, which maintains excellent performance and robustness. It is prolonged when calculating optical flow features. Optical flow is an independent branch of vision and is often formulated as the problem of estimating a 2D projection of the natural 3D motion of the world. The result^27^ shows that optical flow features informative correlations with edges and slight displacements in action recognition is relatively large, so Cai et al. recently used optical flow information and skeleton information as the input of the dual-stream network architecture^28^ and obtained fairly good results. After the deep learning method is applied in this field, the traditional design based on artificial prior knowledge is quickly eliminated by the advantages of learning effective representations on video data without manual feature selection and identifying complex high-level activities^29, 30^. The work in^31^ is to use CNN to extract features based on the IDT algorithm and extend it on the motion trajectory of the features. This method greatly simplifies the feature extraction process and successfully applies CNN to the feature extraction of human actions^32^. Extended the traditional CNN to 3D-CNN with temporal information, performed feature computation in video data’s temporal and spatial dimensions, and connected the feature maps during the convolution process with data in multiple consecutive frames^22^. It uses a multi-resolution CNN to divide the input video into two independent data streams of low resolution and original resolution, perform feature extraction on the two sets of data streams, respectively, and finally combine the results obtained from the two sets of data through Softmax. In the same year^21^, a dual-stream structure fused with spatiotemporal networks was proposed, which takes deep learning a significant step forward in action recognition. The structure divides video information into spatial and temporal data, where space refers to the surface of a single frame, time refers to the optical flow characteristics between frames, CNN implements two different streams, and finally, the results are fused.

### Action recognition based on GCNs method

The above methods almost all process the RGB channel information of the video. The RGB channel information will inevitably introduce much noise and redundant data, harming the recognition results and speed. To weaken the influence of this part, researchers try to extract the skeleton data of the human body as the model’s input. The skeleton sequence does not exist in the form of a traditional Euclidean space, a tree-like topology. It is unreasonable to use CNN to process such a data structure. Yan et al. applied the GCN network to human skeleton-based action recognition for the first time^9^. The ST-GCN network they constructed realizes the dynamic modeling of bones based on the time series of human joint positions and by extending GCN to ST-GCN to capture this spatiotemporal change. Still, the selection of its convolution kernel can only aggregate local features, and the attention mechanism in this work is not very good at finding the association of non-connected joints. Li et al. proposed the AS-GCN network based on it^10^, which uses the classic Encoder-Decoder framework to divide the skeletal data information into (Actional links) A-links and S-links (Structurals links), A-links represent the connection between the specified joint and all other related joints of the body. Compared with the limitation of ST-GCN that only refers to adjacent joints, S-links extend the referenced joints to several adjacent joints to aggregate information on a larger receptive field. The 2S-AGCN network proposed by Shi et al.^11^ transforms the attention mechanism from GCN multiplication to addition and successfully establishes the connection of non-adjacent joints. Based on previous work, Liu et al.^12^ proposed a feature extractor named MS-G3D, which successfully solved the problem of biased weights in multi-scale adjacency matrices while using the adjacency matrix of a single frame to define the connection relationship between joints in adjacent multi-frames through which the complex spatiotemporal joint features between skeleton sequences are captured. The proposed CTR-GCN achieves a breakthrough of GCNs in the field of skeleton-based action recognition by optimizing the skeleton topology with information from different input channels^16^. Skeleton data has advantages in identifying simple actions completed by single or double individuals, it appears weak in identifying group activities due to a lack of background information. But the idea of GCN can still serve as a guiding ideology for identifying group activities. Liu et al.^33^ proposed a visual semantic graph neural network to complete the action recognition task of group activities. It first divides image data into multiple tokens, defines the connection relationship between tokens, and uses GCN to aggregate the connections between different tokens to realize group activity recognition.

### Action recognition based on self-attention mechanism

Self-attention was first proposed^17^. It is a model established to notice keywords in text analysis in the NLP field. Its’ principle is to calculate the inner product between feature vectors to generate a correlation matrix between vectors so that the network can pay more attention to the features with high correlation, which has been popular in the CV field in recent years due to its good portability and superiority^34, 35^. In the field of human action recognition based on skeleton data, to improve the recognition ability of the model, a standard method is to enable the model to accurately find out the spatiotemporal dependence characteristics between the joints of the human body during the movement process. The optimization of the adjacency matrix in section “Action recognition based on GCNs method” is all for this purpose. Plizzari et al.^36^ applied Self-attention to skeleton-based human action recognition for the first time. The proposed ST-TR network framework can more accurately reflect the spatiotemporal dependencies between joints than GCN-based methods. This method will overestimate the relationship between some joints, which will lead to confusion in the overall model parameters, reducing the accuracy of the network. Liu et al.^37^ proposed the KA-AGTN network structure on this basis, the structure uses a multi-head Self-attention to simulate the high-order spatial dependency between joints and uses an adjacency matrix to revise the dependency, which improves the problem of overestimating the relationship between joints in ST-TR, and the proposed TKA module in this structure enhances the correlation of the joints in the temporal dimension, and the whole framework obtains competitive results. Zhao et al.^38^ utilized attentive graph convolutional networks to achieve geometry-aware facial expression recognition. To alleviate the physical design defects of the convolution kernel itself, Zhao et al.^39^ connected the attention template of Adaptive learning with all other positions to achieve feature extraction of a large receptive field.

GCNs focuses on the original physical connections between joints and is a method based on certain prior knowledge; Self-attention focuses on the action connections between joints and is an adaptive relationship between joints, then the model only focuses on a few important joints, making it difficult to disentangle the lightweight relationships between the remaining entangled joints. Our proposed method takes into account the two connection relationships mentioned above and defines the relationship between the two in the form of hyperparameters.

## Method

### Preliminaries

The input action sequence is a series of multi-frame 3D skeleton joint coordinates. Let ${\textbf {I}}$=$\left\{ {\textbf {X}},{\textbf {Y}},{\textbf {Z}}\right\} \in \mathbb {R}^{T\times J\times C}$ be an input skeleton sequence, where ${\textbf {X}}\in \mathbb {R}^{T\times J}$, ${\textbf {Y}}\in \mathbb {R}^{T\times J}$, ${\textbf {Z}}\in \mathbb {R}^{T\times J}$ denote *T* frames of *J* joints with *x*, *y*, and *z* coordinates respectively. *C* represents the number of input channels.

In previous work, people used the method of multi-scale GCN to extract spatial features of skeleton sequences, remarkably, the work^12^ improved the weight bias problem of the adjacency matrix by disentangling neighborhoods, improving the feature extraction ability of GCN. The resulting multi-scale GCN formula is as

1 $$\begin{aligned} \textbf{I}_{(t)}^{(l+1)}=\sigma \left( \sum _{k=0}^{K} \tilde{\textbf{D}}_{(k)}^{-\frac{1}{2}} \tilde{\textbf{A}}_{(k)} \tilde{\textbf{D}}_{(k)}^{-\frac{1}{2}} \textbf{I}_{(t)}^{(l)} \Theta _{(k)}^{(l)}\right) \end{aligned}$$

where $\tilde{\textbf{D}}_{(k)}^{-\frac{1}{2}} \tilde{\textbf{A}}_{(k)} \tilde{\textbf{D}}_{(k)}^{-\frac{1}{2}}$ is the normalized k-adjacency, $\textbf{I}_{(t)}^{(l)}$ represents the feature at layer *l* of the $t_{th}$ frame, $\Theta _{(k)}^{(l)}$denotes a learnable weight matrix at layer *l* of a network and $\sigma (\cdot )$ is an activation function (ReLU).

**Figure 1:**
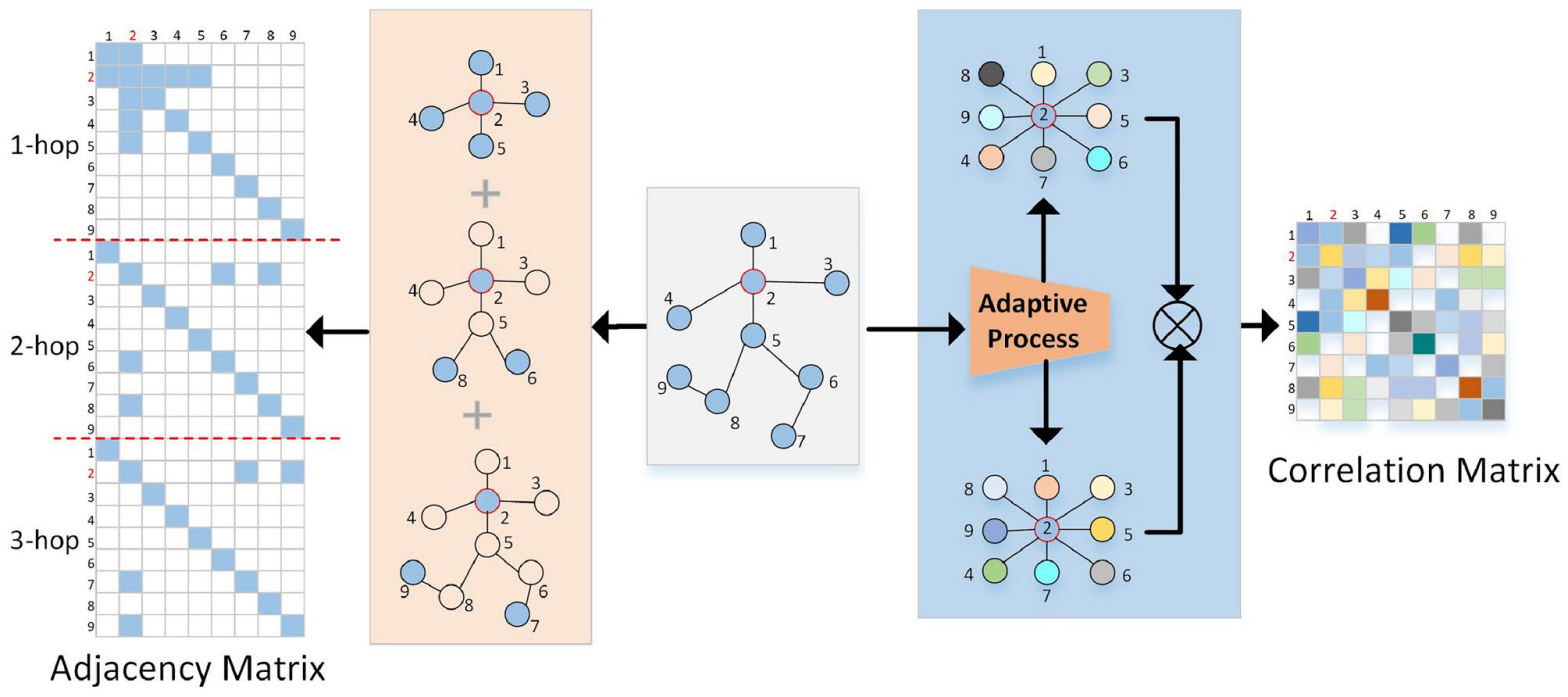
Consider multi-scale feature aggregation for skeleton-based graph structure at node 2 (red). Left: Unbiased weights at the same k-hop and between different k-hops cause indiscriminate feature aggregation. In addition, massive “0” values exist in the matrix obtained by splicing the adjacency matrices of different K hops, resulting in parameter redundancy. Right: In this work, we propose to adjust multi-scale feature aggregation by exploiting an Attention-based strategy, where adaptive processing is used to generate an adaptive correlation matrix, making sure that every node in the correlation matrix gets different correlations at different k-hops(different colors denote nodes’ correlations).

It is not difficult to find from Eq. (1) that the effectiveness of multi-scale GCN is at the cost of parameter redundancy, as each adjacency matrix can only represent one connection relationship. To obtain the dependencies of one joint and others, we must expand the value of *k*. In the NTURGBD dataset and the Kinetics Skeletons dataset, the *k* values are 12 and 8, respectively. At the same time, there is still a problem with the GCNs processing method, which is to artificially assign the exact distance between joints unified to the same value. For example, the *k* value corresponding to the joints at the spine relative to the forehead and the knees is 3, which means the dependency between them is the same. It is unreasonable and will reduce the ability of GCNs to extract features. Empirically, the dependencies between joints are all different. That is, the element values on the adjacency matrix should be different from each other, and GCNs cannot achieve such a condition, however, Self-attention can solve this problem well. Figure 1 shows the advantages and disadvantages of the two methods. The formula of Self-attention^17^ is

2 $$\begin{aligned} {\text {Attention}}({\textbf {Q, K, V}})={\text {Softmax}}\left( \frac{{\textbf {QK}}^T}{\sqrt{d_{k}}}\right) {\textbf {V}} \end{aligned}$$

where **Q, K** and **V** are the data obtained by the input feature **I** through the linear layer, $\sqrt{d_{k}}$ is a constant designed to keep the gradient value of the model stable during the training process, usually taking the number of channels of **Q**, Softmax is to normalize the matrix generated by ${\textbf {QK}}^T.$

We explain the effectiveness of the attention mechanism in the feature extraction from the perspective of vectors. The skeleton sequence of the input model is a 3D matrix-vector of (*C*, *T*, *J*), and three sets of data ${\textbf {Q}}\in \mathbb {R}^{C\times T\times J}$, ${\textbf {K}}\in \mathbb {R}^{C\times T\times J}$ and ${\textbf {V}}\in \mathbb {R}^{C\times T\times J}$ are obtained through three linear layers. In fact, **Q, K** and **V** are all linear mappings of the input data **I**. The reason why **I** is not directly used to replace **Q, K** and **V** in Eq. (2) is to improve the fitting ability of the model. Do not consider channel dimension. ${\textbf {QK}}^T$ can obtain a $J\times J$ matrix.

The element values in the square matrix can physically represent the degree of correlation between row vectors, then $J_{mn}$ represents the degree of correlation between the $m\textrm{th}$ joint and the $n\textrm{th}$ joint. The matrix can achieve the same function as the adjacency matrix in GCNs after it is normalized and the spatial dependencies between the joints are determined. Unlike the adjacency matrix, the elements in the correlation matrix are arbitrary values, indicating that the dependencies between the joints are different and then multiplied with **V** to achieve the purpose of aggregating features.

**Algorithm 1:**
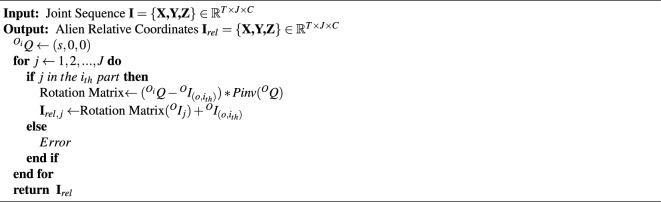
Calculate Alien relative coordinates.

### Data preprocessing (DP)

The key to human action recognition is to extract rich and highly recognizable features from limited data. Therefore, it is considerable to enrich the original data artificially. In this work, the preprocessing module’s data input to the main frame consists of four parts: (1) original data information, (2) velocity information, (3) acceleration information, and (4) alien relative coordinates information.

**Figure 2:**
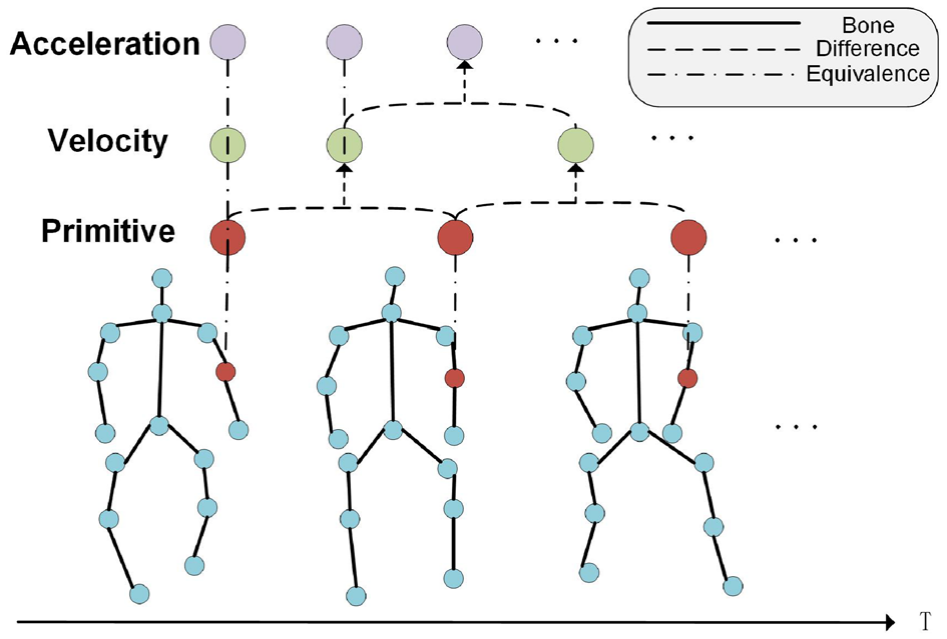
The acquisition of velocity information and acceleration information of skeleton sequence. The original series differentiate the velocity information, and the first frame of the original sequence is taken as the first frame of the velocity information. The speed information distinguishes the acceleration information, and the first two frames are regarded as the first.

**Figure 3:**
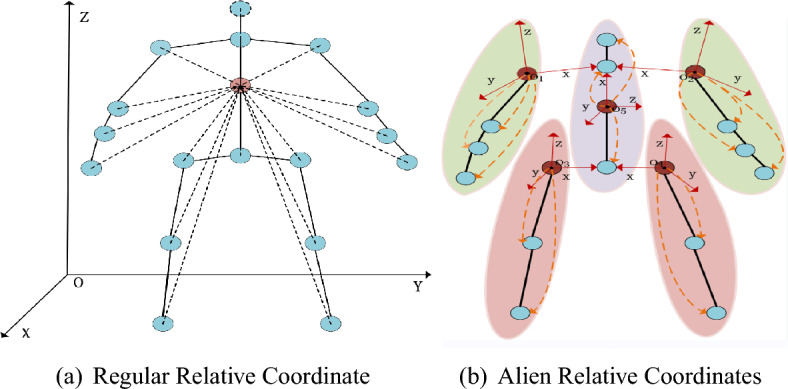
Schematic diagram of the acquisition methods of two different relative coordinates. Left: In the conventional comparative coordinate acquisition method, all joints and the center joint are differentiated. Right: The human body is divided into five parts, each part has it’s center coordinate and coordinate system, and the brown joint is the origin of each piece.

According to the content of section “Preliminaries”, the original human 3D skeleton sequence is expressed as ${\textbf {I}}=\{{\textbf {X, Y, Z}}\}\in \mathbb {R}^{T\times J\times C}$, to calculate the speed information of the sequence, and do the difference operation of subtracting the previous frame from the original motion data. The corresponding calculation formula is

3 $$\begin{aligned} {\textbf {I}}_{vel}={\textbf {I}}[\textrm{t}+1,:,:]-{\textbf {I}}[\textrm{t},:,:] \end{aligned}$$

where t = 1, 2, 3 ...$T-1$, it should be noted that the speed sequence has only $T-1$ frames. To facilitate subsequent calculations, we keep the first frame of the original data. Similarly, to calculate the acceleration information of the sequence, a differential operation can be performed based on the velocity information, and the corresponding calculation formula is

4 $$\begin{aligned} {\textbf {I}}_{\text {acc}}={{\textbf {I}}_{\text {vel}}}[\textrm{t}+1,:,:]-{{\textbf {I}}_{\text {vel}}}[\textrm{t},:,:] \end{aligned}$$

where (t = 2, 3, 4 ..., $T-1$), we also keep the first two frames of the velocity sequence for later processing. The visualization of this part is shown in Fig. 2. Usually, the relative coordinates of the human skeleton^19, 36^ are expressed as ${\textbf {I}}_{\text {rel}}={\textbf {r}}_i(i=1, 2,\ldots , J)$, where

5 $$\begin{aligned} {\textbf {r}}_{\text {i}}={{\textbf {I}}}[:,\textrm{i},:]-{{\textbf {I}}}[:,\textrm{m},:] \end{aligned}$$

where *m* represents the index of the central joint of the human body, as shown in Fig. 3a. However, when analyzing the dataset, we found that the relative positions of some joints and the central joint hardly change during the movement. Physically, it can be considered that these joints perform rigid body motion. That is, simply subtracting the coordinates of these joints from the coordinates of the center joint of the body produces little useful information.

To enrich the data features and reduce the parameter redundancy caused by this time-invariant feature, we design a processing module of ‘alien relative coordinates’. Specifically, we divide the human body into five parts and determine five joints whose relative positions to the central joint were almost unchanged during the movement, as is shown in Fig. 3b.

In order to solve the problem of lack of changes in rotation and scaling mathematically in Eq. (5), we use the central joint of each part as the origin to construct its coordinate system, which benefits from the applications of moving frames in differential geometry^40^.

Taking the part of the left arm as an example. We artificially set the bone between the neck and the left shoulder as the *X* axis and set the coordinates of the joint at the neck to *Q*[*s*, 0, 0], where *s* is a hyperparameter, it represents the scaling relationship of the coordinate transformation. The transformation relationship between the coordinate systems is

6 $$\begin{aligned} \mathrm {{}^{O_i}{} {\textbf {I}}}=\textrm{T}\left[ \mathrm {{}^{O}{} {\textbf {I}}}\right] +\mathrm {R_i} \end{aligned}$$

where ${}^{O}{} {\textbf {I}}$ and ${}^{O_i}{} {\textbf {I}}$ represent the joint coordinates in the *O* and $O_i$ coordinate systems, respectively. $T[\cdot ]$ represents the rotation relationship between the two coordinate systems, and $R_i$ represents the translation relationship between the two coordinate systems. The rotation relationship between the *O* and $O_i$ coordinate systems can be obtained in Eq. (7)

7 $$\begin{aligned} \mathrm {T[\cdot ]}= \left( {}^{O_i}{} {\textbf {I}}-\mathrm {R_i}\right) * {\text {Pinv}}\left[ \mathrm {}^{O}{} {\textbf {I}}\right] \end{aligned}$$

where $Pinv[\cdot ]$ represents the pseudo-inverse operation of the matrix. $T[\cdot ]$ can be calculated by substituting the coordinates of point *Q* in the *O* coordinate system and the $O_i$ coordinate system, then the coordinates of the rest of the joints of the $i_{th}$ part in the $O_i$ coordinate system can be calculated, the algorithm flow of this method is shown in Alg. 1, where ${ }^{O}I_{(o, i_{th})}$ represents the coordinates of the origin of the $i_{th}$ part in the *O* coordinate system. We connect the above four sets of information in the channel dimension, and Eq. (8) can represent the entire data preprocessing module.

8 $$\begin{aligned} {\textbf {I}}_{DP}=BN(Concat({\textbf {I}}, {\textbf {I}}_{rel}, {\textbf {I}}_{vel}, {\textbf {I}}_{acc})) \end{aligned}$$

where ${\textbf {I}}_{DP}$, ${\textbf {I}}$, ${\textbf {I}}_{rel}$, ${\textbf {I}}_v$, ${\textbf {I}}_{acc}$ represent output data, original data, alien relative coordinates information, velocity information, and acceleration information, respectively. BN represents the BatchNorm layer.

### Spatial feature extraction module (SFEM)

**Figure 4:**
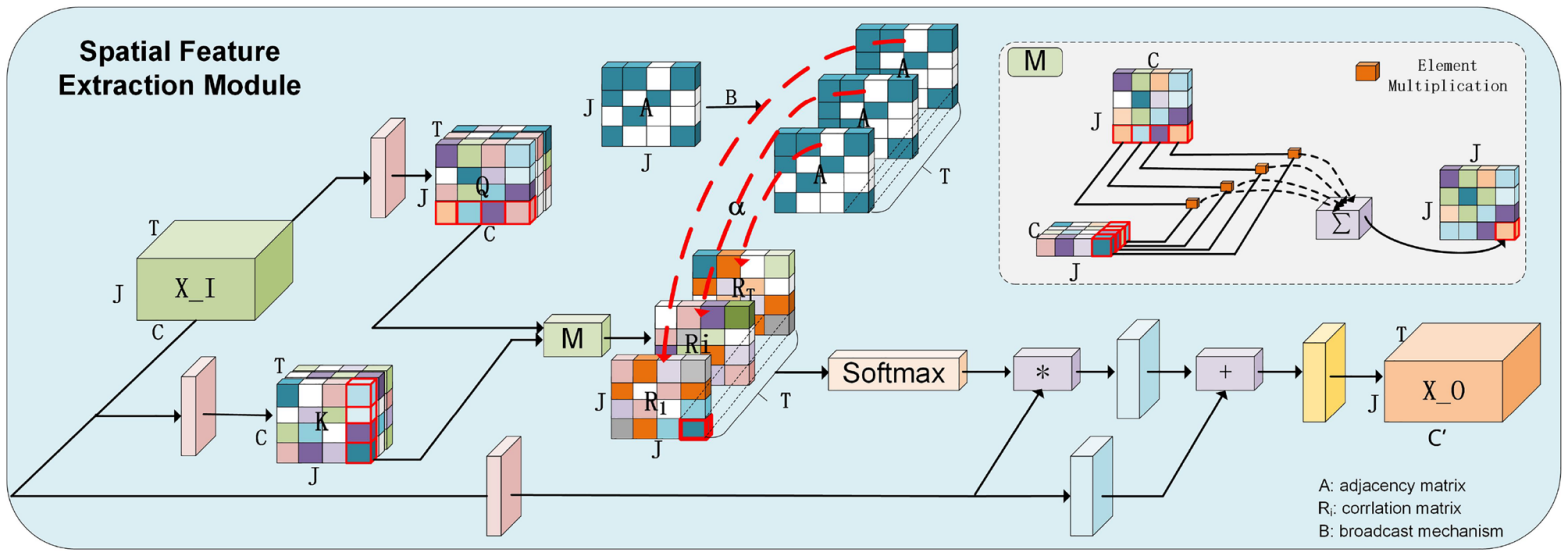
Spatial feature extraction module. Pink blocks represent linear layers, blue blocks represent convolutional layers, and yellow blocks represent BN and activation layers. Q, K means the input data through the linear layer, the same size as the original data, and M represents the way to obtain the inner product between vectors (red). The red dotted line represents the corresponding addition of the element values of the adjacency matrix to the correlation matrix obtained by Self-attention. $\alpha$ is a hyperparameter, it represents the weighting coefficient between the adjacency matrix and the correlation matrix.

As a model with competitive results at present, MS-G3D^12^ uses the multi-scale GCN method to represent the non-connected relationship in the process of skeleton action, according to the content of section “Preliminaries”, there are two problems in this processing method that need to be improved: the amount of computation is *k* times that of a single-scale GCN, where *k* represents the number of scales, and the other is that at the same scale, all joints have the same correlation. Considering the above two problems, we integrate GCNs and the self-attention mechanism to improve it, as illustrated in Fig. 4.

The channel dimension feature is used as the feature vector of the joint in the original self-attention mechanism, and the inner product of the feature vector can be used to obtain a correlation matrix of size $J\times {J}$. There are *T* correlation matrices, which means that each frame has a corresponding spatial connection relationship. Instead of using one correlation matrix to represent the connection of all frames, it is in line with the physical meaning, then the correlation matrix is multiplied by the input features, and the spatial features of the skeleton sequence can be obtained. Finally, a trainable convolutional layer is used to correct the extracted spatial features, the mathematical description is as follows:

9 $$\begin{aligned} {\textbf {I}}_{(t)}^{(l+1)}=\sigma \left( \text{ Softmax }\left( \frac{{\textbf {Q}}_{(t)}^{(l)} {\textbf {K}}_{(t)}^{(l)}}{\sqrt{d m}}\right) {\textbf {V}}_{(t)}^{(l)} \Theta ^{(l)}\right) \end{aligned}$$

where $\textbf{Q}_{(t)}^{(l)}$, $\textbf{K}_{(t)}^{(l)}$, $\textbf{V}_{(t)}^{(l)}$ represent the feature at layer *l* of the $t_{th}$ frame, It is not difficult to find that Self-attention can well express the action connection relationship between the joints in the process of model training, and the distance of them does not limit the correlation between the joints, it can be any value.

This method can improve the problems caused by multi-scale GCN, at the same time, since there is no physical connection between the feature vectors, this way cannot specifically consider the structural connection between the joints. To take into account the inherent structural connection between the joints, we use the broadcasting mechanism to copy the adjacency matrix *T* times and add the adjacency matrix to the obtained correlation matrix to obtain a new correlation matrix. Finally, the input data is multiplied by the new correlation matrix after going through the linear layer, and then a residual structure is added to obtain the final spatial feature. The entire spatial feature extraction module can be represented by Eq. (10)

10 $$\begin{aligned} {\textbf {I}}\_{\textbf {S}}_{(t)}^{(l+1)}=\sigma \left( \text{ Softmax }\left( {\frac{\textbf{Q}_{(t)}^{(l)} {\textbf{K}_{(t)}^{(l)}}}{\sqrt{d m}}}+\alpha {\text{\AA }}\right) {\textbf{V}_{(t)}^{(l)}} \Theta ^{(l)}+ {\textbf{V}_{(t)}^{(l)}}\right) \end{aligned}$$

where Å=$\tilde{\textbf{D}}^{-\frac{1}{2}}{\mathbf {(A + I)}} \tilde{\textbf{D}}^{-\frac{1}{2}}$, $\alpha$ is a hyperparameter, it represents the weighted relationship between the correlation matrix obtained by Self-attention and the adjacency matrix inherent in the GCN. $\Theta ^{(l)}$denotes a learnable weight matrix at layer *l* of the network and $\sigma (\cdot )$ is an activation function, the residual connection of this module is realized by $\textbf{V}_{(t)}^{(l)}$.

### Temporal feature extraction module (TFEM)

**Figure 5:**
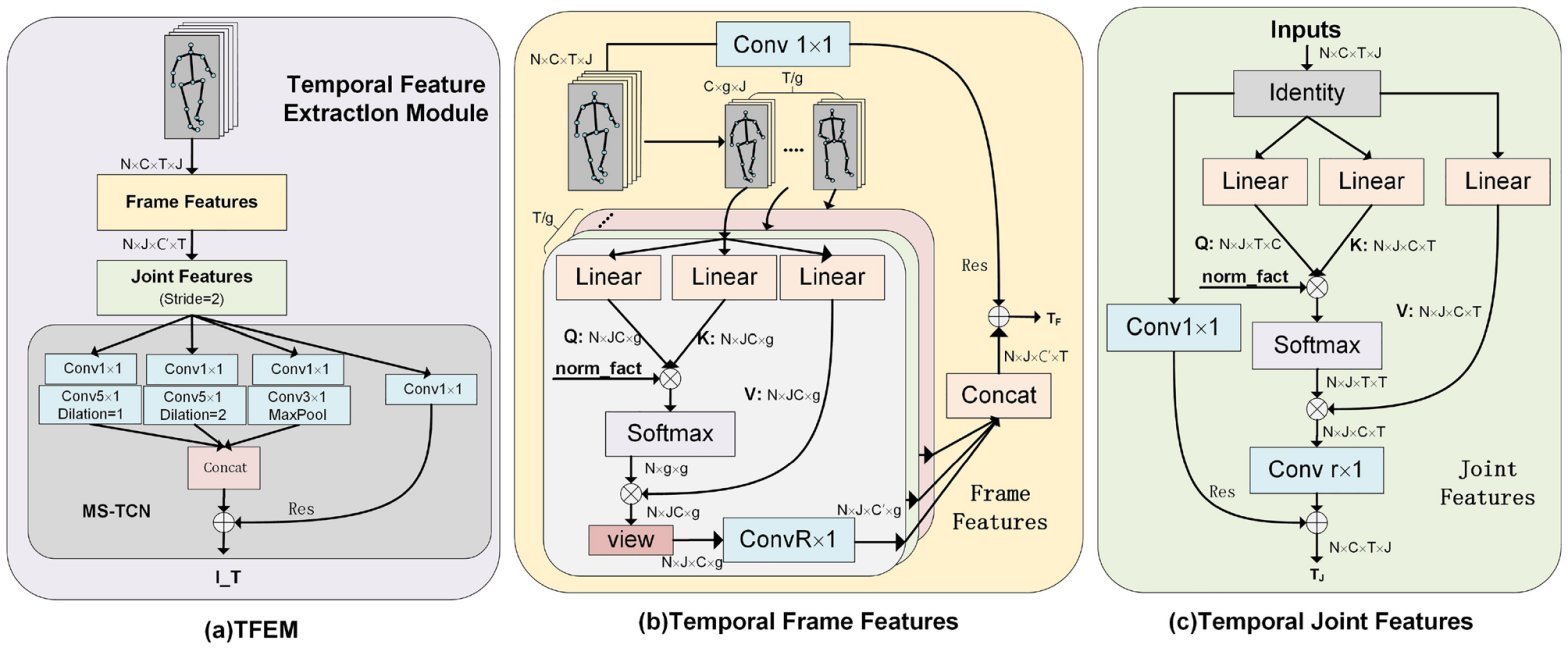
(**a**) Temporal feature extraction module. It is composed of Frames Features (FF), Joint Features (JF), and MS-TCN in series. JF has stride = 1 in the first block and stride = 2 in the rest. (**b**) Frames Features (FF). *Linear* represents the Linear layer, and *view* represents the dimension transformation function of the data. *Concat* indicates that the data is connected in the time dimension. (**c**) Joint Features(JF). *Identity* represents a placeholder function. $Conv x\times x$ represents a two-dimensional convolutional layer with a convolution kernel of $x\times x$. *g*, *R*, *r* are all hyperparameters.

The frames of the human skeleton sequence have the characteristics of linear arrangement, which allows researchers to directly use the convolution method to aggregate the information of *r* frames to extract the temporal features between the skeleton sequences. The most commonly used method is TCN^41^, which uses a convolution kernel of dimension $r\times 1$ to aggregate the information of one joint in *r* frames. Then this way does not have the ability the model to capture the characteristic changes of the joints over a long period. It is necessary to preprocess the feature relationships between all frames to alleviate this problem before performing local information aggregation. Naturally, we analogize the attention mechanism used in section “Spatial feature extraction module (SFEM)”, as shown in Fig. 5.

Cause it is to obtain the feature changes of the joints between different frames, according to Eq. (2), *J* correlation matrices of size $T\times T$ are calculated first, each matrix represents the correlation of a joint in different frames, then multiply these *J* correlation matrices with the original sequence to get the characteristics of the joints in the long-term distance. Based on this feature, we use a convolutional layer with a kernel dimension of $r\times 1$ to extract the features of the joints over a short temporal distance, as shown in Fig. 5c. The mathematical description of this part is as follows:

11 $$\begin{aligned} {\textbf {T}}_{J}^{(l+1)}=\sigma \left( \text{ Softmax }\left( \frac{{\textbf {Q}}_{(j)}^{(l)} {\textbf {K}}_{(j)}^{(l)}}{\sqrt{d m}}\right) {\textbf {V}}_{(j)}^{(l)} \Theta ^{(l)} + res\right) \end{aligned}$$

where $\textbf{Q}_{(j)}^{(l)}$, $\textbf{K}_{(j)}^{(l)}$, $\textbf{V}_{(j)}^{(l)}$ represent the feature at the layer *l* of the $j\textrm{th}$ joint. The dimensions of Softmax$[\cdot ]$ is [N, J, T, T], j = 0,1,..., J−1. The above feature extraction is considered from the point of view of joints. Generally, humans follow the principle of first whole and then part when recognizing actions, that is, take the frame as a whole to find the relatively important frame first and then process the joint information in the frame, while most of the previous works ignore the necessity of holistic consideration. In this module, we use the attention mechanism to realize the overall consideration of temporal features, as shown in Fig. 5b.

Since the features of each frame are expressed in the form of a matrix with a size of $J\times C$, and the matrix cannot be calculated for the inner product, it needs to be converted into a one-dimensional vector, we slice the matrix with the joint dimension, and splices the vectors obtained from the slices to obtain a one-dimensional vector with a dimension of $JC\times 1$. Let this vector as the feature vector of each frame, then do the inner product calculation to obtain the correlation matrix between frames, since the information based on the frame as a whole is relatively general, it will inevitably cause redundancy of information and reduce the accuracy of the model if the correlation of all frames is calculated. In this part, we let *g* frames as a group to calculate the correlation between *g* frames, *g* is a hyperparameter. The results of the *T*/*g* groups are spliced together as the extracted temporal features. The mathematical expression of temporal feature extraction taking the frame as a whole is shown in Eq. (12).

12 $$\begin{aligned} \textbf{T}_{F}^{(l+1)}=\sigma \left( \text{ Concat } \left( \text{ Softmax }\left( \frac{\textbf{Q}_{i}^{(l)} \textbf{K}_{i}^{(l)}}{\sqrt{d m}}\right) \textbf{V}_{i}^{(l)} \right) \Theta ^{(l)} + res\right) \end{aligned}$$

where the dimensions of ${\textbf {Q}}_{i}^{(l)}$, ${\textbf {K}}_{i}^{(l)}$, and ${\textbf {V}}_{i}^{(l)}$ are all [*N*, *C*, *g*, *J*], *i*=$0, 1,\ldots , T/g-1$. *Concat* indicates that the results of *g* groups are concatenated in the time dimension. The whole temporal feature extraction module is composed of Frame Features (FF), Joint Features (JF) and TCN in series, as shown in Fig. 5a.

### Spatio-temporal feature extraction module (STFEM)

In SFEM and TFEM, we extract the spatial and temporal features of the skeleton sequence, respectively. From the principle analysis in sections “Spatial feature extraction module (SFEM)” and “Temporal feature extraction module (TFEM)”, it can be seen that these two modules are separated from each other. That is, they cannot capture complex spatio-temporal associations. Empirically, the spatio-temporal features between joints can provide a lot of adequate information for human action recognition task. In G3D^12^, the author artificially determined the connection relationship between joints in consecutive $\tau$ frames by designing a spatio-temporal adjacency matrix, as shown in Eq. (13), and realizes the spatio-temporal feature modeling of joints.

13 $$\begin{aligned} {A}_\tau =\left[ \begin{array}{ccc} {A} &{} \cdots &{} {A} \\ \vdots &{} \ddots &{} \vdots \\ {A} &{} \cdots &{} {A} \end{array}\right] \in R^{\tau J \times \tau J} \end{aligned}$$

However, a single frame still determines the connection between joints in this multi-frame. Due to the limitation of the adjacency matrix of the joints, more generalized modeling cannot be achieved. In the original paper, the author used a multi-scale method to alleviate this problem and achieved specific results, while it also increased the computational load of the model. Inspired by this work and the principle in section “Spatial feature extraction module (SFEM)”, we use Self-attention to construct a correlation matrix across space and time and complete the modeling of spatio-temporal features of skeleton sequences.

In this module, we first calculate the correlation matrix of all joints in the $\tau$ frame, and concatenate the $\tau$ adjacency matrices representing the joint connection relationship in a single frame, then add the calculated correlation matrix to the concatenated adjacency matrix, in addition, the hyperparameter $\beta$ is also used to control the proportion of the adjacency matrix. The entire spatio-temporal feature extraction module can be represented by Eq. (14)

14 $$\begin{aligned} \left[ \mathbf {I\_{F}}_{(\tau )}^{(l+1)}\right] _t=\sigma \left( {\text {Softmax}}\left( {\frac{\left[ \textbf{Q}_{(\tau )}^{(l)}\right] _t \left[ \textbf{K}_{(\tau )}^{(l)}\right] _t}{\sqrt{d m}}}+\beta {\text{\AA }}_\tau \right) \left[ \textbf{V}_{(\tau )}^{(l)}\right] _t \Theta ^{(l)}+res\right) \end{aligned}$$

where $\tau$ represents the joint relationship in consecutive $\tau$ frames, $\left[ \textbf{Q}_{(\tau )}^{(l)}\right] _t$, $\left[ \textbf{K}_{(\tau )}^{(l)}\right] _t$, $\left[ \textbf{V}_{(\tau )}^{(l)}\right] _t$ represent the feature of the [t,t+$\tau$] frames in $l_{th}$ layer through the linear layer, t=0, 1,..., $T-1$, all data whose index *i* exceeds *T* are filled with “0”.

### Overall framework

**Figure 6:**
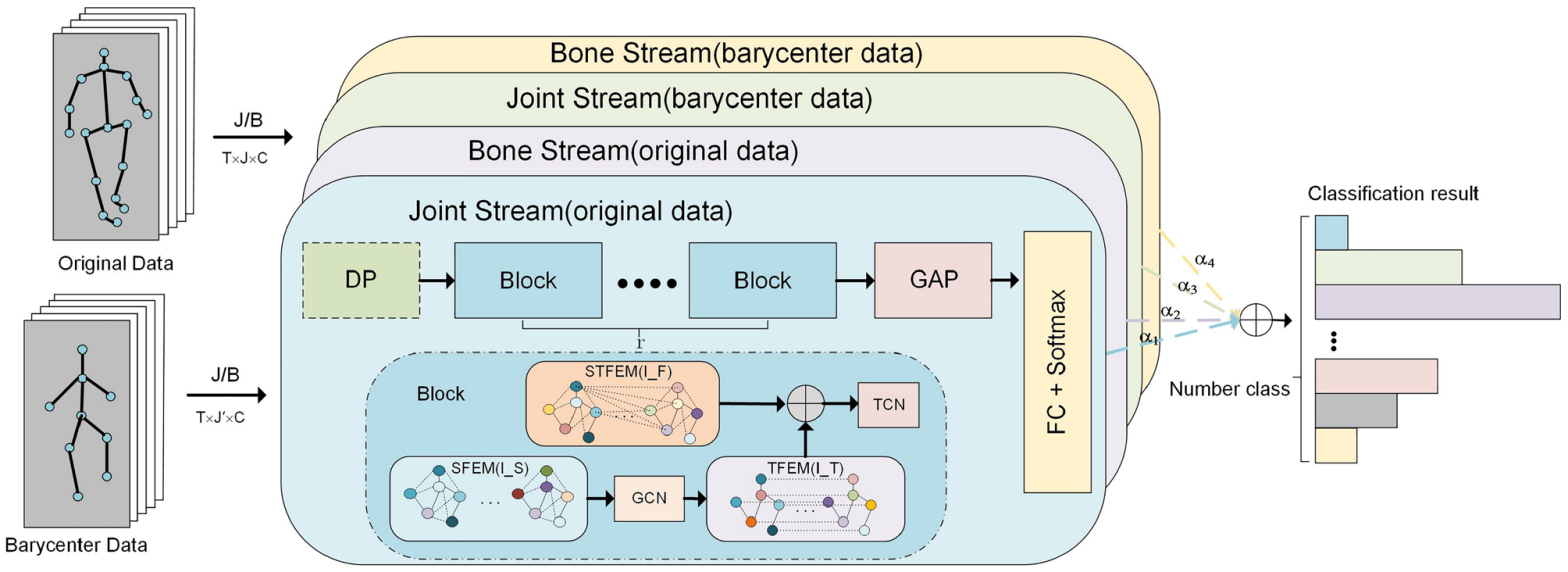
The overall stream of our frame. *DP* represents the data preprocessing module, which only appears in an original data stream. *r* indicates the number of basic blocks in each stream, $r=3$ here. *GAP* and *FC* represent the Global Average Pooling and Fully Connected, respectively, $\alpha _{i}(i=1,2,3,4)$ indicates the proportion of the results of different streams in the overall framework, $\oplus$ represents the addition of the corresponding element values in the matrix.

Inspired by the dual-stream network^42^ and the idea of multi-scale^43^, the overall framework of this paper uses the multi-scale dual-stream network shown in Fig. 6. Specifically, the dual-stream network representation uses joint and bone data to train separate models with the same architecture, respectively, and weights the softmax scores from the joints/bones to get the final prediction score. The bone feature is obtained by differentiating the adjacent joints far from the center of the body. It should be noted that to ensure that the bone and joint features can use the same architecture, we add a zero bone vector in the center of the human body so as to obtain *J* bones from *J* joins, and an adjacency matrix is used to define the connection relationship.

We found in the experiments that using the original joint calibration method will make the data lack the generalizing information, which is beneficial to the task of human motion recognition. So we use multi-scale to perform the fusion operation on the original data as shown in Fig. 6. That is, the barycenter of multiple adjacent joint data is taken as the generalized information (mean value) of this part, which will be input into the model for training. Finally, the softmax score and the softmax score obtained at the original scale are weighted and summed to obtain the final score.

## Experiments

We conduct experiments to verify the effect of the proposed model on three large-scale skeleton action recognition datasets of NTU-RGB+D 60^44^, NTU-RGB+D 120^45^ and Kinetics-Skeleton^46^.

### Datasets

*NTU-RGB+D 60* NTU-RGB+D 60 is a large-scale motion data set open-sourced by the Rose Laboratory of Nanyang Technological University, Singapore in 2016. The dataset contains 60 types of actions, of which 40 categories are daily behavior actions, 9 categories are health-related actions, and the remaining 11 categories are two-person interactive actions. All actions are performed by 40 individuals. The 3D joint data includes the three-dimensional space coordinates of J = 25 joint points (x, y, z). It uses two division criteria when dividing the training set and the test set. (1) Cross-Subject (X-Sub): the training set and the test set are divided according to the person ID. The training set has 40,320 samples, and the test set has 16,560 samples. (2) Cross-View (X-View): the training set and the test set are divided according to the camera. The 18960 samples collected by camera 1 are used as the test set, and 37920 collected by cameras 2 and 3 as the training set.

*NTU-RGB+D 120* NTU-RGB+D 120 extends 60 categories based on NTU-RGB+D 60. The expanded data set has a total of 114480 samples. Same as NTU-RGB+D 60, there are erroneous samples in the NTU-RGB+D 120 dataset, and the effective number of samples after excluding them is 113945. The author replaces Cross-View in NTU-RGB+D 60 with CrossSetup (X-Set) in this dataset, the samples collected from half of the cameras are used as the training set (54468 samples in total), and the rest are used as the test set; in Cross-Subject, half of the samples collected by 106 volunteers were selected for the training set (a total of 63,026 samples), and the rest was used for the test set.

*Kinetics Skeleton 400* The Kinetics Skeleton 400 dataset is further extracted based on the Kinetics 400 video dataset. It contains more than 400 types of skeleton samples. There are 240436 samples in the training set and 19796 samples in the test set. Each sample includes J = 18 joint, the feature of each joint is composed of its 2D space coordinates and OpenPose’s prediction confidence score. Meanwhile, the upper limit of the number of skeletons in each frame time is 2.

### Implementations

Unless otherwise stated, all models have r = 3 and are trained with SGD with momentum 0.9, the loss function is Cross Entropy, batch size 32 (16 per worker), an initial learning rate of 0.05 (can linearly scale up with batch size^21^) for 50, 60, and 65 epochs with step LR decay with a factor of 0.1 at epochs (30, 40), (30, 50), and (45, 55) for NTU-RGB+D 60, 120, and Kinetics Skeleton 400, respectively. The corresponding interactions are 1260/1184 (Sub/View), 1602/1968 (Sub/Set), and 7512, respectively. Weight decay is set to 0.0005 for final models and is adjusted accordingly during component studies. All skeleton sequences are padded to T = 300 frames by replaying the actions. Our model was implemented using the PyTorch deep learning framework and the experiments were conducted on two RTX(3090, 24GB) GPUs.

### Comparison against the state of the arts

We compare the proposed AMANet with the current SOTA models on the NTU60, NTU120, and Kinetics-Skeleton 400 datasets. The results of the comparison are shown in Table 1, where we classify these SOTA models into three types, namely early traditional methods, GCN-based methods, and Self-attention-based methods. According to these experiment results, we have three observations as follows:

- Compared with the current SOTA model, the traditional methods have absolute disadvantages: HCN is only 86.5$\%$ and 91.1$\%$ on X-Sub and X-View in NTU 60, ST-LSTM is 55.7$\%$ and 57.9$\%$ in NTU 120, which are far lower than the accuracy of GCN-based method and Self-attention-based method.
- GCN-based models outperform attention-based models: As a classic model-based GCN, the accuracy of MS-G3D reaches 91.5$\%$ and 96.2$\%$ in NTU 60, 86.9$\%$ and 88.4$\%$ in NTU 120. In contrast, the performance of the Self-attention method-based model is not promising. For example, the accuracy of KA-AGTN^11^ is 90.4$\%$ and 96.1$\%$ in NTU 60, 86.1$\%$ and 88.0$\%$ in NTU 120.
- Our method shows the best results compared with other works: AMANet gets the accuracy of 92.1$\%$ and 96.6$\%$ in NTU 60, 86.7$\%$ and 88.6$\%$ in NTU 120. In Kinetics 400, our model is 0.2$\%$ and 0.1$\%$ higher than MS-G3D and KA-AGTN under the standard of Top1 and is 0.3$\%$ and 0.1$\%$ higher than them under the standard of Top5, which reaches the level of SOTA in this dataset.

**Table 1:** Comparisons of the recognition accuracy ($\%$) with the state-of-the-art methods on the NTU-RGB+D 60, NTU-RGB+D 120, and Kinetics Skeleton 400 datasets.

Methods	NTU-RGB+D 60		NTU-RGB+D 120		Kinetics Skeleton 400	
	X-Sub($\%$)	X-View($\%$)	X-Sub($\%$)	X-Set($\%$)	Top-1($\%$)	Top-5($\%$)
Ind-RNN^47^	81.8	88.8	–	–	–	–
HCN^48^	86.5	91.1	–	–	–	–
PA-LSTM^44^	–	–	25.5	26.3	–	–
ST-LSTM^49^	–	–	55.7	57.9	–	–
ST-GCN^9^	81.5	88.3	70.7	73.2	30.7	52.8
2S-AGCN^11^	88.5	95.1	82.9	84.9	36.1	58.7
SGN^50^	89.0	94.5	79.2	81.5	–	–
AGC-LSTM^51^	89.2	95.0	–	–	–	–
DGNN^18^	89.9	96.1	–	–	36.9	59.6
DC-GCN+ADG^52^	90.8	96.6	86.5	88.1	–	–
HSR-TSL^53^	87.7	94.4	–	–	–	–
MS-G3D^12^	91.5	96.2	**86.9**	88.4	38.0	60.9
EfficientGCN^20^	91.7	95.7	–	–	–	–
Tripool^54^	88.0	95.3	90.0	96.7	34.1	56.2
Ta-CNN^55^	90.4	94.8	85.4	86.8	–	–
Ta-CNN+^55^	90.7	95.7	85.7	87.3	–	–
ST-TR^36^	89.9	96.2	85.1	87.1	33.6	56.1
KA-AGTN^37^	90.4	96.1	86.1	88.0	38.1	61.1
AMANet	**92.1**	**96.6**	86.7	**88.6**	**38.2**	**61.2**

The best results are shown in bold.

According to the defect that the adjacency matrix in the original GCN can only express the explicit relationship between joints, the researchers improved the adjacency matrix so that it can aggregate the implicit relationship between joints to a certain extent, then this improvement is often accompanied by parameter redundancy. As a method in the NLP field, the text data processed by the Self-attention has no explicit and implicit differences, so it has a congenital disadvantage when migrating it to the skeleton data, however, it cannot be denied that it has high research value in this field. Based on the analysis above, we aim to integrate GCNs and Self-attention with the proposed AMANet, the accuracy of which outperform MS-G3D, KA-AGTN by the margin of 0.6$\%$ and 0.4$\%$, and 1.7$\%$ and 0.5$\%$ in NTU 60, − 0.2$\%$ and 0.2$\%$, and 0.6$\%$ and 0.6$\%$ in NTU 120, respectively. We believe that the main reason why AMANet is not effective in the X-Sub of the NTU120 dataset is that AMANet is modified on the basis of the framework built for GCN, and the integration of attention in this framework cannot fully reflect the ability of AMANet to extract features, but this does not affect the feasibility of this fusion method in action recognition tasks.

### Ablation study

In this section, we verify the effectiveness of the various parts proposed in section “Method”, including DP, SFEM, TFEM, STAF, and MS-DS. The baseline is selected as the performance of MS-G3D on the Cross-Subject setting of the NTU-RGB+D 60 dataset.

**Figure 7:**
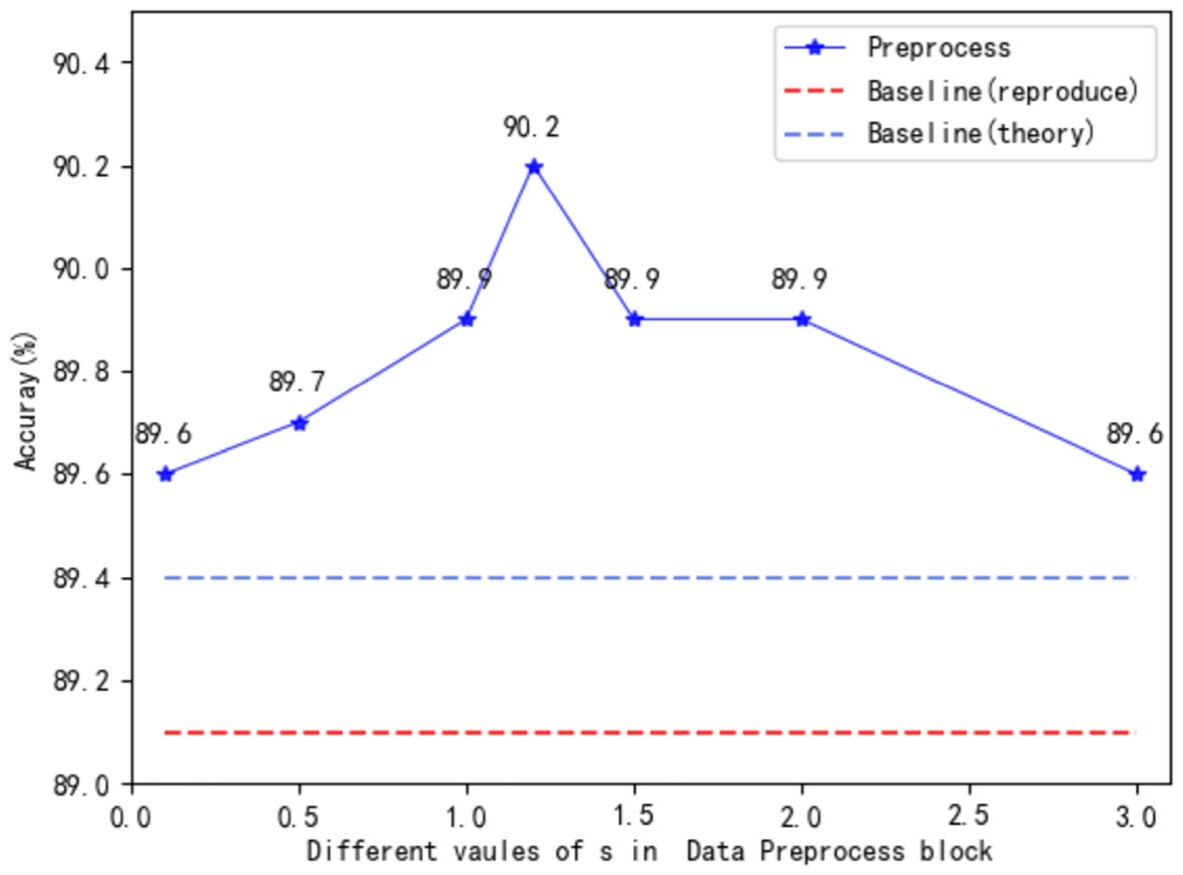
Visual comparison of different values of s in Data Preprocessing block. Whether it is compared with the reproduced results or the theoretical results in the paper, the baseline after adding the DP module can achieve higher accuracy.

*DP*: To illustrate the effectiveness and generalization ability of this module, we directly add this module on the basis of the baseline (JS), as shown in Table 2. Based on the original data, when the velocity and acceleration information are added, the baseline can be improved by 0.6$\%$, and coupled with the general relative coordinate information, the model can achieve a 0.8$\%$ improvement. When the general relative coordinate information is replaced with the alien relative coordinate information designed in section “Data preprocessing (DP)”, the model can achieve a 1.1$\%$ improvement. When obtaining the relative coordinate information, the hyperparameter *s* represents the scaling relationship between the original data and the relative coordinate information, which greatly affects the accuracy of the model. We compare the accuracy of the model under different s values in Fig. 7. It can be seen from the results in the figure that the accuracy of the model shows an approximately symmetrical distribution with the change of *s*, the accuracy of the model is the highest when *s* is 1.2. We also input the general relative coordinate information and relative coordinate information into the model at the same time, the results show that the result of this method is 0.3$\%$ lower than adding only the relative coordinate information, indicating that there is a conflict between the two kinds of information, which can interfere with the training of the model.

**Table 2:** Comparisons of the recognition accuracy ($\%$) of the model obtained by adding different input information in the data preprocessing module. W(x) means adding x information to the module.

Model configurations	Input channels	ACC($\%$)
Baseline(MS-G3D)	3	89.1
W(v, a)	9	89.7
W(v, a, ori_rel)	12	89.9
W(v, a, ali_rel)	12	**90.2**
W(v, a, ori_rel, ali_rel)	15	89.9

The best results are shown in bold.

**Figure 8:**
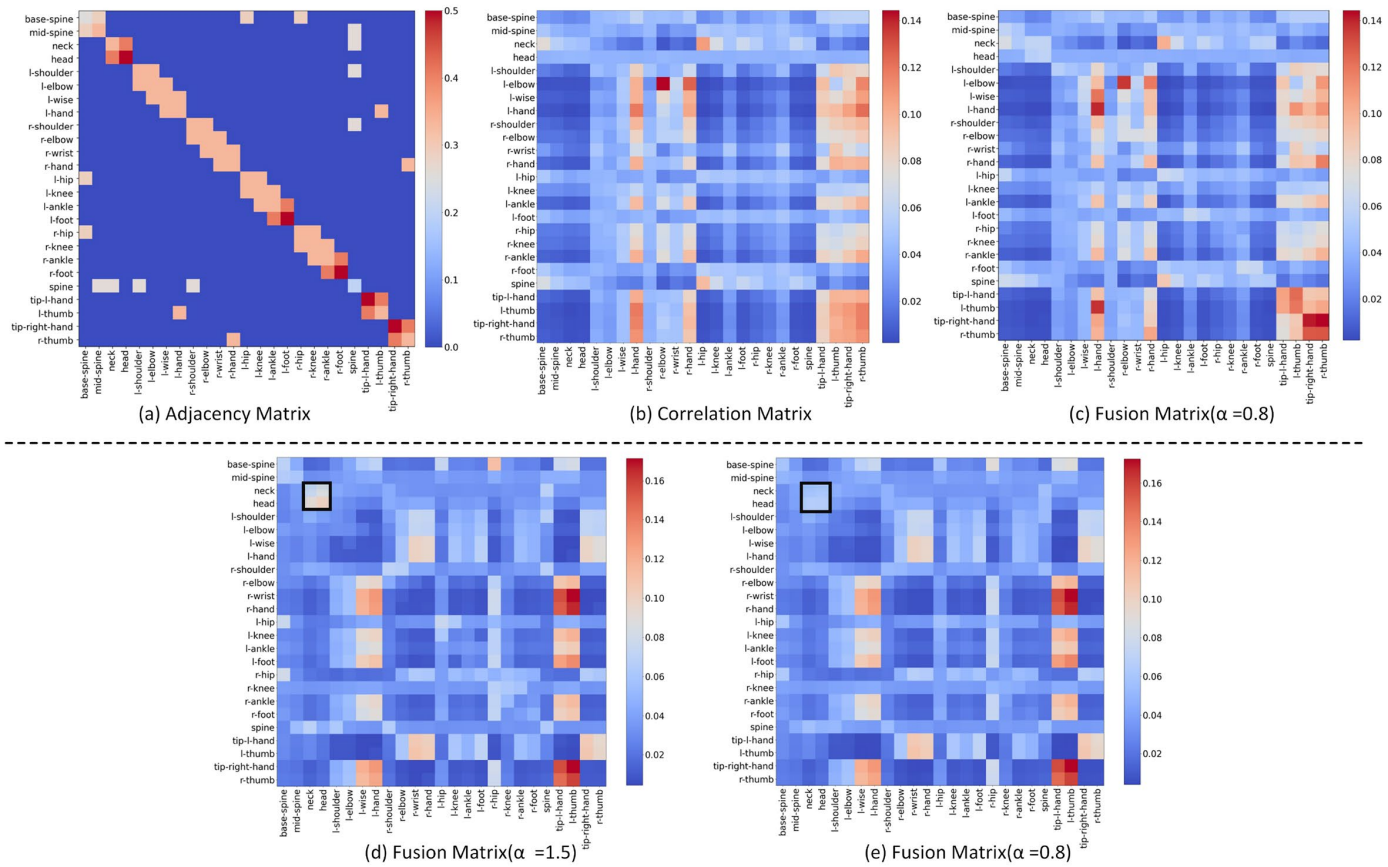
Visualized validation. The upper and bottom represent “clapping” and “watching time”, respectively. (**a**) Normalized adjacency matrix of skeleton graph corresponding to NTURGBD dataset. (**b**) The trained correlation matrix outputted by the last layer of the AMANet($\alpha =0$). (**c**) Matrix after the fusion of adjacent and correlation matrix($\alpha =0.8$). (**d**) Fusion matrix($\alpha =1.5$). (**e**) Fusion matrix($\alpha =0.8$).

*SFEM* We have two baselines in this part of the ablation experiment, one is to make the spatial scale k = 1 in MS-G3D(JS), and the other k = 13. The experimental results are shown in Tables 3 and 4. To prove the correlation matrix can better reflect the connection between the joints than the initial adjacency matrix, we replace the MS-GCN (k = 1) with STEM, the model accuracy is improved by 0.4$\%$ when $\alpha$ is 0.8, but it is 0.3$\%$ lower than the baseline when k = 13, indicating that the multi-scale method is still very competitive. When we add SFEM directly in front of MS-GCN, the accuracy of the model reaches 89.5$\%$, which is 0.4$\%$ higher than that of using MS-GCN only, and the model increases the parameters by 0.1 M, indicating that the Self-attention can make the model pay attention to features ignored by GCNs methods. The visualization results in the upper of Fig. 8 also support the above conclusion.

**Table 3:** Replace MS-GCN in baseline with SFEM.

$\alpha$	0.1	0.2	0.5	0.8	1	1.5
Acc(%)	88.2	88.6	88.4	**88.8**	88.4	88.3

The best results are shown in bold.

Comparing the accuracy of the model with different $\alpha$ values.

**Table 4:** Compare the accuracy of the model when GCN, MS-GCN and SFEM are used as spatial feature extractors respectively.

Model configurations	Params	FLOPs	ACC($\%$)
Baseline (k = 1)	2.4**M**	24.04**G**	88.4
GCN-> SFEM	2.8**M**	25.79**G**	**88.8**
Baseline (k = 13)	3.2**M**	29.95**G**	89.1
MS-GCN->SFEM$+$MS-GCN	3.3**M**	32.17**G**	**89.5**

The best results are shown in bold.

*TFEM* In order to demonstrate the effectiveness of the modules in Temporal feature extraction module (TFEM), we conducted the experiment as shown in Table 5. In baseline, the time feature extraction module is composed of two MS-TCNs in series. We first replace one of them with the Joint-Based Feature(JF) extraction module, which improves the accuracy of the model by 0.2$\%$, indicating that it is effective to conduct a preliminary attention analysis of the entire skeleton sequence before the short-range feature extraction of joints. Then we replace one of MS-TCNs with the Frame-Based Feature (FF) extraction module, which improves the accuracy of the model by 0.3$\%$, indicating that grouping operation can avoid information interference between long-distance frames when all joints in a frame are treated as a whole. Finally, in the form of Fig. 5, we concatenate the three modules into a whole, which improves the accuracy of the model by 0.5$\%$. There are three hyperparameters in this module, *r*, *R*, and *g*, where *r* means to use the convolution with dimension $r\times 1$ to extract the time characteristics of consecutive *r* frames in the skeleton sequence. In TFEM, we utilize MS-TCN to complete this work, therefore, $r=1$ here. From the principle analysis in section “Temporal feature extraction module (TFEM)”, we divide the skeleton sequence into *T*/*g* groups and extract the features between the g-frames data in each group. Since the information in frame units is relatively vague, only the features of the short-term distance are extracted. Naturally, we take the same value for the hyperparameter *g*, which represents the number of frames per group, and the hyperparameter *R*, which represents the number of aggregated short-term distance frames, that is, $g=R$. According to the results in Table 5, when $g=5$, the accuracy of the model reaches 89.6$\%$, which is 0.5$\%$ higher than the baseline, while the number of parameters of the model is only increased by 0.6M.

**Table 5:** Replace MS-TCN in baseline with TFEM.

Model configurations	Params	FLOPs	ACC($\%$)
Baseline(MS-G3D)	3.2**M**	29.95**G**	89.1
2 MS-TCNs->JF+MS-TCN	3.1**M**	32.07 **G**	89.3
2 MS-TCNs->FF+MS-TCN	3.3**M**	34.19 **G**	89.4
2 MS-TCNs->TFEM(g = 3)	3.7**M**	37.41 **G**	89.4
2 MS-TCNs->TFEM(g = 5)	3.8**M**	39.23 **G**	**89.6**
2 MS-TCNs->TFEM(g = 7)	4.0**M**	41.86 **G**	89.3

The best results are shown in bold.

Compare the accuracy of the model under various settings. *a*->*b* means *a* replaces with *b*.

*STFEM* The experimental results of this part are shown in Tables 6 and 7. Like SFEM, there are two baselines in this part of the ablation experiment, one is to set the spatiotemporal scale k=1 in G3D, and the other is k = 5. First, in order to prove that the correlation matrix can better reflect the spatiotemporal joint relationship between skeleton sequences than the adjacency matrix, we replace the G3D (k = 1) module in the baseline with STFEM. When the hyperparameter $\beta$ is set to 0.8, the accuracy of the model is improved by 0.2$\%$, but 0.4$\%$ lower than the baseline (k = 5). At the same time, when the hyperparameter is too small ($\beta$ = 0.1) or too large ($\beta$ = 1.5), the accuracy of the model is low, indicating that the importance of the inherent connection relationship between the joints and the relationship obtained by the correlation matrix is relatively balanced. Then we added STFEM directly to the front of the G3D module, and the model accuracy was 89.3$\%$, which was 0.2$\%$ higher than using G3D alone, indicating that the relationship between the skeleton sequences obtained by using the correlation matrix can complement the joint relationship represented by the adjacency matrix.

**Table 6:** Compare the accuracy of the model when using G3D, MS-G3D, and STFEM as feature extractors for spatio-temporal fusion respectively.

Model configurations	Params	FLOPs	ACC($\%$)
Baseline (k = 1)	2.4**M**	18.12**G**	88.5
G3D-> STFEM	1.8**M**	14.93 **G**	**88.7**
Baseline (k = 5)	3.2**M**	29.95**G**	89.1
MS-G3D-> STFEM$+$MS-G3D	3.4**M**	36.26**G**	**89.3**

The best results are shown in bold.

**Table 7:** Replace MS-G3D in baseline with STFEM.

$\beta$	0.1	0.2	0.5	0.8	1	1.5
Acc (%)	88.2	88.4	88.6	**88.7**	88.2	88.0

The best results are shown in bold.

Comparing the accuracy of the model with different $\beta$ values.

**Figure 9:**
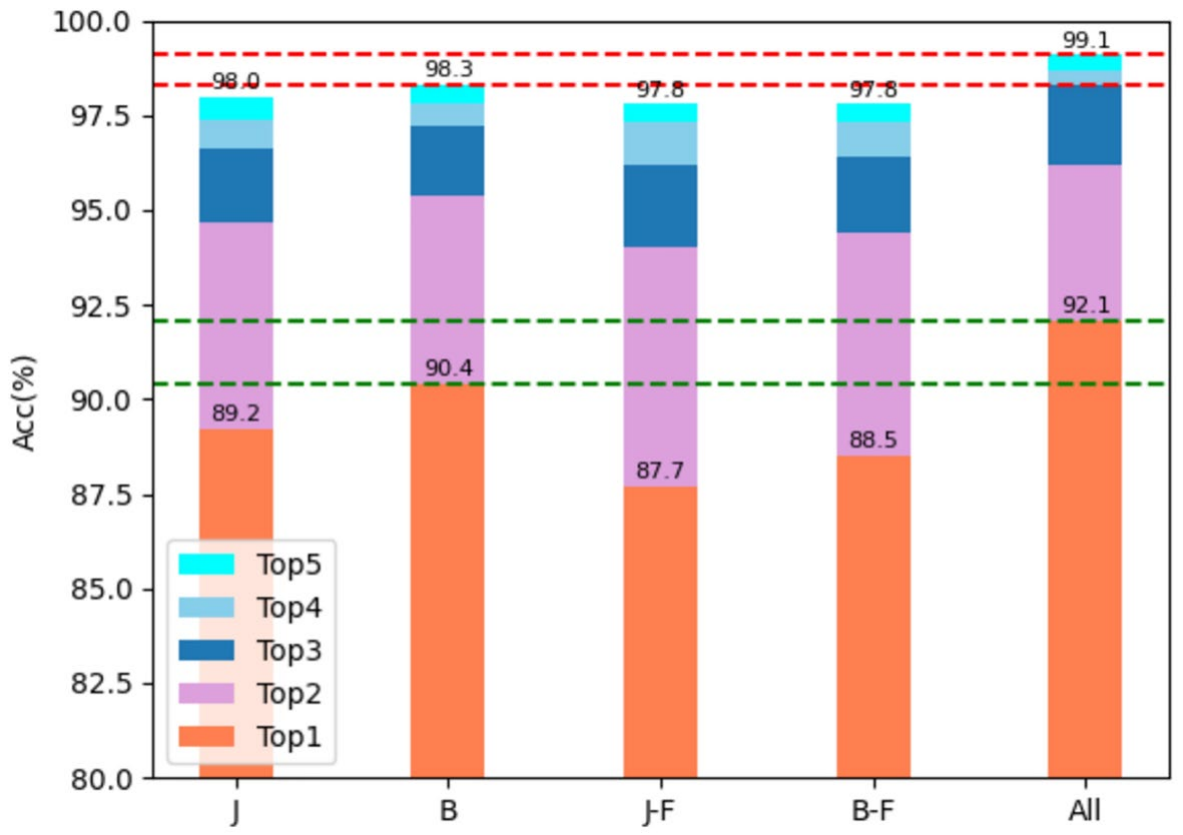
Visualization of the accuracy (Top1−Top5) of various streams on the X-Sub of NTU60 dataset. J-F and B-F represent fused barycenter data of joints and bones, respectively.

*MS-DS* As shown in Fig. 9, the dual-stream architecture is to add the softmax results obtained from different data correspondingly, so that the score changes from the previous non-first to the first, and the accuracy is improved. As can be seen from Table 8, the results obtained only from joint data and bone data are 89.2$\%$ and 90.4$\%$, respectively, and the weighted summation can obtain an accuracy of 91.7$\%$. Coupled with the results obtained by the barycenter data proposed in section “Overall framework”, the accuracy of the entire model reaches 92.1$\%$, which is highly competitive in the current SOTA.

**Table 8:** Compare the accuracy of different streams of the model on the X-Sub of NTU60 dataset.

Method	Joint	Bone	JF	BF	Acc($\%$)
Baseline(JS)	$\surd$				89.4
Baseline(BS)		$\surd$			90.1
Baseline(2S)	$\surd$	$\surd$			91.5
AMANet(J-FS)			$\surd$		87.7
AMANet(B-FS)				$\surd$	88.5
AMANet(JB-FS)			$\surd$	$\surd$	89.7
AMANet(JS)	$\surd$				89.2
AMANet(BS)		$\surd$			90.4
AMANet(JBS)	$\surd$	$\surd$			91.7
AMANet(A11)	$\surd$	$\surd$	$\surd$	$\surd$	92.1

*Order ablation* AMANet has three different modules to extract spatial (SFEM), temporal(TFEM), and spatiotemporal entangled features (STFEM), among which the SFEM and TFEM are sequential. As mentioned by Woo et al.^56^: As each module has different functions, the order may affect overall performance. We changed the order connection relationship between SFEM and TFEM to obtain the overall performance, the results in Table 9 shows that when extracting spatial features first and then temporal features, the overall model’s various indicators are more advanced than the reverse.

**Table 9:** The impact of different orders of SFEM and TFEM on model accuracy.

Model configurations	Params	FLOPs	ACC($\%$)
Baseline	3.2**M**	29.95 **G**	89.1
SFEM-TFEM	4.3**M**	44.23 **G**	**89.5**
TFEM-SFEM	4.7**M**	46.93 **G**	89.3

The best results are shown in bold.

a–b represents the data flow from a to b.

### Limitation analysis

In SFEM, we exploit the adjacency matrix in GCN and the correlation matrix in self-attention to characterize the physical connection and motion connection between joints respectively, and utilize hyperparameter $\alpha$ to determine the weight relationship between the two matrices. Intuitively, it will make the model adaptively fit the optimal value when taking A as a trainable parameter. But we found in the ablation study that when $\alpha$ is treated as a hyperparameter with a value of 0.8, the model will achieve better results.

As (1) the weights of the physical and motion connections between different joints are variable in an action: In “clap hands”, the focus of the model is on the motion connections of the hand joints, where the weight of their physical connections is relatively weak, but other joints still require physical connections to maintain the basic skeleton structure. (2) the value of $\alpha$ should also be variable in different actions: In “watch time”, in addition to the action connections of the hand joints, fine-grained movements of the head and neck joints are equally important. But when selecting the same $\alpha$-value as “clapping” for this action, it is inevitable that the model will ignore (strengthen) the proportion of relevant features. Merely, the dataset currently used is more suitable for the situation when $\alpha$= 0.8. The comparison between the upper and bottom of Fig. 8 characterizes this limitation

## Conclusion

In this paper, we present an Adaptively Multi-correlations Aggregation Network (AMANet) for improving skeleton-based action recognition. Firstly, a data preprocessing module (DP) is designed for skeleton data enhancement. Then, we design three key modules: the Spatial Feature Extraction Module (SFEM) to capture motion dependencies in the spatial-wise channels, Temporal Feature Extraction Module (TFEM) to exploit temporal information for all motion joints, and the Spatio-Temporal Feature Extraction module (STFEM) to comprehensively model motion dynamics. Extensive experiments have proven the effectiveness of AMANet.

However, the integration of Self-attention has not brought a significant improvement to AMANet since it does not fully utilize the powerful feature extraction ability of Self-attention. In future work, we will seek to build a framework that conforms to its mechanism according to this fusion method, so that the attention-based methods and GCNs-based methods can stimulate each other for better performance improvement.

## Data Availability

The data that support the findings of this study are openly available at http://rose1.ntu.edu.sg/Datasets/actionRecognition.asp.

## References

[1] Rautaray SS, Agrawal A (2015). Vision based hand gesture recognition for human computer interaction: A survey. Artif. Intell. Rev..

[2] Chakraborty BK, Sarma D, Bhuyan MK, MacDorman KF (2018). Review of constraints on vision-based gesture recognition for human-computer interaction. IET Comput. Vis..

[3] Fujiyoshi H, Hirakawa T, Yamashita T (2019). Deep learning-based image recognition for autonomous driving. IATSS Res..

[4] Chen L (2020). Survey of pedestrian action recognition techniques for autonomous driving. Tsinghua Sci. Technol..

[5] Khurana, R. & Kushwaha, A. K. S. Deep learning approaches for human activity recognition in video surveillance-a survey. In *2018 First International Conference on Secure Cyber Computing and Communication (ICSCCC)* 542–544 (IEEE, 2018).

[6] Tsakanikas V, Dagiuklas T (2018). Video surveillance systems-current status and future trends. Comput. Electr. Eng..

[7] Newell, A., Yang, K. & Deng, J. Stacked hourglass networks for human pose estimation. In *European Conference on Computer Vision* 483–499 (Springer, 2016).

[8] Chen, Y. *et al.* Cascaded pyramid network for multi-person pose estimation. In *Proceedings of the IEEE Conference on Computer Vision and Pattern Recognition* 7103–7112 (2018).

[9] Yan, S., Xiong, Y. & Lin, D. Spatial temporal graph convolutional networks for skeleton-based action recognition. In *Thirty-second AAAI Conference on Artificial Intelligence* (2018).

[10] Li, M. *et al.* Actional-structural graph convolutional networks for skeleton-based action recognition. In *Proceedings of the IEEE/CVF Conference on Computer Vision and Pattern Recognition* 3595–3603 (2019).

[11] Shi, L., Zhang, Y., Cheng, J. & Lu, H. Two-stream adaptive graph convolutional networks for skeleton-based action recognition. In *Proceedings of the IEEE/CVF Conference on Computer Vision and Pattern recognition* 12026–12035 (2019).

[12] Liu, Z., Zhang, H., Chen, Z., Wang, Z. & Ouyang, W. Disentangling and unifying graph convolutions for skeleton-based action recognition. In *Proceedings of the IEEE/CVF Conference on Computer Vision and Pattern Recognition* 143–152 (2020).

[13] Ji X, Zhao Q, Cheng J, Ma C (2021). Exploiting spatio-temporal representation for 3d human action recognition from depth map sequences. Knowl.-Based Syst..

[14] Yin P, Ye J, Lin G, Wu Q (2021). Graph neural network for 6d object pose estimation. Knowl.-Based Syst..

[15] Qiu, J. *et al.* Proceedings of the 24th acm sigkdd international conference on knowledge discovery & data mining - kdd ’18 - deepinf. In *ACM Press the 24th ACM SIGKDD International Conference - London, United Kingdom (2018.08.19-2018.08.23)* 2110–2119 (2018).

[16] Chen, Y. *et al.* Channel-wise topology refinement graph convolution for skeleton-based action recognition. In *Proceedings of the IEEE/CVF International Conference on Computer Vision* 13359–13368 (2021).

[17] Vaswani A (2017). Attention is all you need. arXiv.

[18] Shi, L., Zhang, Y., Cheng, J. & Lu, H. Skeleton-based action recognition with directed graph neural networks. In *Proceedings of the IEEE/CVF Conference on Computer Vision and Pattern Recognition* 7912–7921 (2019).

[19] Song, Y. F., Zhang, Z., Shan, C. & Wang, L. Richly activated graph convolutional network for robust skeleton-based action recognition. In *IEEE Transactions on Circuits and Systems for Video Technology* **PP** 1–1 (2020).

[20] Song, Y.-F., Zhang, Z., Shan, C. & Wang, L. Constructing stronger and faster baselines for skeleton-based action recognition. In *IEEE Transactions on Pattern Analysis and Machine Intelligence* (2022). 10.1109/TPAMI.2022.3157033 35254974

[21] Feichtenhofer, C., Pinz, A. & Zisserman, A. Convolutional two-stream network fusion for video action recognition. In *Proceedings of the IEEE conference on computer vision and pattern recognition* 1933–1941 (2016).

[22] Wei, S.-E., Ramakrishna, V., Kanade, T. & Sheikh, Y. Convolutional pose machines. In *Proceedings of the IEEE Conference on Computer Vision and Pattern Recognition* 4724–4732 (2016).

[23] Le, Q. V., Zou, W. Y., Yeung, S. Y. & Ng, A. Y. Learning hierarchical invariant spatio-temporal features for action recognition with independent subspace analysis. In *CVPR 2011* 3361–3368 (IEEE, 2011).

[24] Donahue, J. *et al.* Long-term recurrent convolutional networks for visual recognition and description. In *Proceedings of the IEEE Conference on Computer Vision and Pattern Recognition* 2625–2634 (2015). 10.1109/TPAMI.2016.2599174 27608449

[25] Wang H, Kläser A, Schmid C, Liu C-L (2013). Dense trajectories and motion boundary descriptors for action recognition. Int. J. Comput. Vis..

[26] Wang, H. & Schmid, C. Action recognition with improved trajectories. In *Proceedings of the IEEE International Conference on Computer Vision* 3551–3558 (2013).

[27] Sevilla-Lara, L. *et al.* On the integration of optical flow and action recognition. In *German Conference on Pattern Recognition* 281–297 (Springer, 2018).

[28] Cai, J., Jiang, N., Han, X., Jia, K. & Lu, J. Jolo-gcn: Mining joint-centered light-weight information for skeleton-based action recognition. In *Proceedings of the IEEE/CVF Winter Conference on Applications of Computer Vision* 2735–2744 (2021).

[29] Wang, L. *et al.* Temporal segment networks: Towards good practices for deep action recognition. In *European Conference on Computer Vision* 20–36 (Springer, 2016).

[30] Lan, Z., Zhu, Y., Hauptmann, A. G. & Newsam, S. Deep local video feature for action recognition. In *Proceedings of the IEEE Conference on Computer Vision and Pattern Recognition Workshops* 1–7 (2017).

[31] Wang, L., Qiao, Y. & Tang, X. Action recognition with trajectory-pooled deep-convolutional descriptors. In *Proceedings of the IEEE Conference on Computer Vision and Pattern Recognition* 4305–4314 (2015).

[32] Ji S, Xu W, Yang M, Yu K (2012). 3d convolutional neural networks for human action recognition. IEEE Trans. Pattern Anal. Mach. Intell..

[33] Liu T, Zhao R, Lam K-M, Kong J (2022). Visual-semantic graph neural network with pose-position attentive learning for group activity recognition. Neurocomputing.

[34] Li C (2021). Memory attention networks for skeleton-based action recognition. IEEE Trans. Neural Netw. Learn. Syst..

[35] Liu, R. *et al.* Attention mechanism exploits temporal contexts: Real-time 3d human pose reconstruction. In *Proceedings of the IEEE/CVF Conference on Computer Vision and Pattern Recognition* 5064–5073 (2020).

[36] Plizzari C, Cannici M, Matteucci M (2021). Spatial Temporal Transformer Network for Skeleton-Based Action Recognition.

[37] Liu Y, Zhang H, Xu D, He K (2022). Graph transformer network with temporal kernel attention for skeleton-based action recognition. Knowl.-Based Syst..

[38] Zhao R, Liu T, Huang Z, Lun DP-K, Lam KK (2021). Geometry-aware facial expression recognition via attentive graph convolutional networks. IEEE Trans. Affect. Comput..

[39] Zhao, H. *et al.* Psanet: Point-wise spatial attention network for scene parsing. In *Proceedings of the European Conference on Computer Vision (ECCV)* 267–283 (2018).

[40] Nakova G (2015). Moving frames and differential forms: From Euclid past Riemann by Jose G. Vargas. Differ. Geom. Phys. Math..

[41] Zhou, B., Andonian, A., Oliva, A. & Torralba, A. Temporal relational reasoning in videos. In *Proceedings of the European Conference on Computer Vision (ECCV)* 803–818 (2018).

[42] Xu Q (2021). Scene image and human skeleton-based dual-stream human action recognition. Pattern Recogn. Lett..

[43] Shi, L., Zhang, Y., Cheng, J. & Lu, H. Two-stream adaptive graph convolutional networks for skeleton-based action recognition. In *Computer Vision and Pattern Recognition* (2018). 10.1109/TIP.2020.3028207 33035162

[44] Shahroudy, A., Liu, J., Ng, T.-T. & Wang, G. Ntu rgb+ d: A large scale dataset for 3d human activity analysis. In *Proceedings of the IEEE Conference on Computer Vision and Pattern Recognition* 1010–1019 (2016).

[45] Liu J (2019). Ntu rgb+ d 120: A large-scale benchmark for 3d human activity understanding. IEEE Trans. Pattern Anal. Mach. Intell..

[46] Kay, W. *et al.* The kinetics human action video dataset. arXiv:1705.06950 (2017).

[47] Li, S., Li, W., Cook, C., Zhu, C. & Gao, Y. Independently recurrent neural network (indrnn): Building a longer and deeper rnn. In *Proceedings of the IEEE Conference on Computer Vision and Pattern Recognition* 5457–5466 (2018).

[48] Li, C., Zhong, Q., Xie, D. & Pu, S. Co-occurrence feature learning from skeleton data for action recognition and detection with hierarchical aggregation. arXiv:1804.06055 (2018).

[49] Liu, J., Shahroudy, A., Xu, D. & Wang, G. Spatio-temporal lstm with trust gates for 3d human action recognition. In *European conference on computer vision* 816–833 (Springer, 2016).

[50] Zhang, P. *et al.* Semantics-guided neural networks for efficient skeleton-based human action recognition. In *Proceedings of the IEEE/CVF Conference on Computer Vision and Pattern Recognition* 1112–1121 (2020).

[51] Si, C., Chen, W., Wang, W., Wang, L. & Tan, T. An attention enhanced graph convolutional lstm network for skeleton-based action recognition. In *Proceedings of the IEEE/CVF Conference on Computer Vision and Pattern Recognition* 1227–1236 (2019).

[52] Cheng, K. *et al.* Decoupling gcn with dropgraph module for skeleton-based action recognition. In *European Conference on Computer Vision* 536–553 (Springer, 2020).

[53] Si C, Jing Y, Wang W, Wang L, Tan T (2020). Skeleton-based action recognition with hierarchical spatial reasoning and temporal stack learning network. Pattern Recogn..

[54] Peng W, Hong X, Zhao G (2021). Tripool: Graph triplet pooling for 3d skeleton-based action recognition. Pattern Recogn..

[55] Xu, K., Ye, F., Zhong, Q. & Xie, D. Topology-aware convolutional neural network for efficient skeleton-based action recognition. In *Proceedings of the AAAI Conference on Artificial Intelligence, vol. 36* 2866–2874 (2022).

[56] Woo, S., Park, J., Lee, J. & Kweon, I. Cbam: Convolutional block attention module. arXiv:1807.06521 (2018).

